# Serum Immunoglobulin G Is Associated With Decreased Risk of Pancreatic Cancer in the Swedish AMORIS Study

**DOI:** 10.3389/fonc.2020.00263

**Published:** 2020-02-28

**Authors:** Sam Sollie, Aida Santaolalla, Dominique S. Michaud, Debashis Sarker, Sophia N. Karagiannis, Debra H. Josephs, Niklas Hammar, Goran Walldius, Hans Garmo, Lars Holmberg, Ingmar Jungner, Mieke Van Hemelrijck

**Affiliations:** ^1^Translational Oncology & Urology Research (TOUR), School of Cancer and Pharmaceutical Sciences, King's College London, London, United Kingdom; ^2^Department of Public Health and Community Medicine, Tufts University School of Medicine, Boston, MA, United States; ^3^Department of Epidemiology, Brown University School of Public Health, Providence, RI, United States; ^4^Department of Medical Oncology, Guy's and St Thomas' NHS Trust, London, United Kingdom; ^5^St John's Institute of Dermatology, School of Basic and Medical Biosciences, King's College London, Guy's Hospital, London, United Kingdom; ^6^Unit of Epidemiology, Institute of Environmental Medicine, Karolinska Institutet, Stockholm, Sweden; ^7^Unit of Cardiovascular Epidemiology, Institute of Environmental Medicine, Karolinska Institutet, Stockholm, Sweden; ^8^Clinical Epidemiological Unit, Department of Medicine, Karolinska Institutet and CALAB Research, Stockholm, Sweden

**Keywords:** serum immunoglobulin, pancreatic cancer, IgA, IgG, IgM, immune system and cancer, AMORIS

## Abstract

**Background:** Emerging evidence points to potential roles of the humoral immune responses in the development of pancreatic cancer. Epidemiological studies have suggested involvement of viral and bacterial infections in pancreatic carcinogenesis. Experimental studies have reported high expression levels of antigens in pancreatic cancer cells. Therefore, we aimed to investigate the role of different components of humoral immunity in the context of pancreatic cancer. We evaluated associations between pre-diagnostic serum markers of the overall humoral immune system [immunoglobulin A (IgA), immunoglobulin G (IgG) and immunoglobulin M (IgM)], and the risk of pancreatic cancer in the Swedish Apolipoprotein-related MORtality RISk (AMORIS) study.

**Methods:** We selected all participants (≥20 years old) with baseline measurements of IgA, IgG or IgM (*n* = 41,900, 136,221, and 29,919, respectively). Participants were excluded if they had a history of chronic pancreatitis and individuals were free from pancreatic cancer at baseline. Multivariate Cox proportional hazards regression was used to estimate risk of pancreatic cancer for medical cut-offs of IgA, IgG, and IgM.

**Results:** Compared to the reference level of 6.10–14.99 g/L, risk of pancreatic cancer was elevated among those with IgG levels <6.10 g/L [HR: 1.69 (95% CI 0.99–2.87)], and an inverse association was observed among those with IgG levels ≥15.00 g/L [0.82 (95% CI 0.64–1.05); *P*trend = 0.027]. The association appeared to be stronger for women than men [HR: 0.64 (95% CI 0.43–0.97) and 0.95 (95% CI 0.69–1.29), respectively]. No associations were observed with IgA or IgM.

**Conclusion:** An inverse association was observed between pre-diagnostic serum levels of IgG and risk of pancreatic cancer. Our findings highlight the need to further investigate the role of immune response in pancreatic cancer etiology.

## Introduction

Pancreatic cancer is predicted to be the second leading cause of cancer-related death by 2030 (1), and is frequently diagnosed at an advanced stage. To date, the etiology of pancreatic cancer is not well understood (2). Apart from smoking (3), long-standing diabetes (4), obesity and chronic pancreatitis (5), little is known about the risk factors and biological processes that lead to pancreatic cancer development (6, 7).

Recently, there has been mounting evidence that the humoral immune system plays a role in the development of pancreatic cancer. Epidemiological studies have suggested that viral and bacterial infections contribute to pancreatic cancer pathogenesis (8–13). Most studies focused on humoral responses to *Helicobacter pylori* and found contradicting results (14–17). More recently, periodontal disease, caused by bacterial infections of pathogens such as *Porphyromonas gingivalis* and *Fusobacterium species*, has been suggested to play a role in the development and prognosis of pancreatic cancer (18–24). Furthermore, other responses of the humoral immune system, such as allergic reactions, may present a protective effect for pancreatic cancer diagnosis (25–29).

In addition to the epidemiological studies, experimental studies have reported a number of antigens that are highly expressed in pancreatic cancer cells (30, 31). For example, significant levels of immunoglobulin (Ig) G antibodies have been detected in cancer patients' plasma, including in pancreatic cancer patients. More specifically, anti-MUC1 serum IgG levels and IgG serum IgG4 concentrations (>135 mg/dL) have been measured in patients with pancreatic cancer (32–34). In contrast, no data is available for other antibodies such as IgA and IgM in relation with pancreatic cancer.

Despite the growing evidence for a role of the humoral immune system in pancreatic cancer development, to our knowledge no epidemiological studies have yet evaluated the association between pre-diagnostic serum markers of the overall humoral immune response and risk of pancreatic cancer. A better understanding of the etiology and underlying biological mechanisms for pancreatic cancer may improve our ability to identify high risk individuals and improve early detection.

We therefore present the first large population-based prospective cohort study to examine pre-diagnostic markers of the overall humoral immune response (IgG, IgA, and IgM) in relation to pancreatic cancer.

## Materials and Methods

### Study Population and Data Collection

The Swedish Apolipoprotein-related MORtality RISk (AMORIS) cohort includes information from blood and urine samples for 812,073 subjects obtained and analyzed between 1985 and 1996. All laboratory analyses were conducted at the Central Automation Laboratory (CALAB), Stockholm. The subjects were residents of Sweden and were predominantly living in the Stockholm county, ranging in age from less than 20 to over 80 years old. All participants were either healthy individuals referred for clinical laboratory testing as part of health check-ups or outpatients referred for laboratory testing (35). A more detailed description of the AMORIS cohort is given elsewhere (35–39).

The AMORIS cohort has been followed via record linkage using the Swedish 10-digit personal identity number in Swedish national health registers, registers of quality of care, and surveys including socio-economic data as well as a questionnaire and biomedical data from number of research cohorts (35). For the purpose of the current study, the National Cancer Register, the Patient Register, the Cause of death Register and the consecutive Swedish Censuses during 1970–1990 have been utilized. This study complied with the Declaration of Helsinki and was approved by the Ethics Review Board of the Karolinska Institutet.

This study included all individuals aged 20 years or older who were free from pancreatic cancer at baseline, as registered in the National Cancer Register dating back to 1958. Furthermore, participants were excluded if they had a history of chronic pancreatitis, as defined in the National Patient Register going back nationally to 1987 and regionally to 1964. All subjects were required to have a baseline measurement of IgA or IgG or IgM measured at a health examination between 1985 and 1996. If a participant had multiple measurements of an immunoglobulin, the first measurement was included in the study to allow for consistency across the entire cohort. Follow-up time was defined as time from baseline measurement until date of cancer diagnosis, death, emigration, or end of the study (31st of December 2011), whichever occurred first.

The main outcome variable was a diagnosis of pancreatic cancer (International Classification of Diseases (ICD), Revision 7 (1955) code 157). We also included the following information from the AMORIS study: serum IgA (g/L), serum IgG (g/L), serum IgM (g/L), serum glucose (mmol/L), age at baseline measurement and gender. From the other registries, we collected information regarding education, Charlson Comorbidity Index (CCI) and socioeconomic status (SES).

The quantitative determination of IgA, IgG, and IgM was performed using turbidimetry with reagents (DAKO – Glostrup, Denmark) using a HITACHI 911 automatic analyser (Boehringer – Mannheim, Germany) with a coefficient of variation <5% (IgA), ≤ 5% (IgG), and ≤ 7% (IgM) (40–42).

### Data Analyses

We estimated the risk of pancreatic cancer with multivariate Cox proportional hazards regression for medical cut-offs used in the CALAB laboratory of IgG (<6.10, 6.10–14.99, ≥15.00 g/L) (41). IgA was dichotomised as <3.66 g/L and ≥3.66 g/L instead of the medical cut-offs (<0.70, 0.70–3.65, ≥3.66 g/L) due to the small number of participants with low IgA levels (40, 43). Levels of IgM were dichotomised as <1.40 g/L and ≥1.40 g/L, as proposed by the normal laboratory values for blood, plasma and serum from the MSD manual (44).

Multivariate Cox proportional hazards regression models were adjusted for age, gender, education, CCI and serum glucose level (continuous variable). A test for trend was conducted by using assignment to medical cut-offs as an ordinal scale for IgG. To assess reverse causation, a sensitivity analysis was conducted in which those who had a follow-up time <1 year and <3 years, respectively, were removed.

With regards to IgG, we performed stratified analyses for age (<55 and ≥55), gender (male and female) and serum glucose levels (<7.00 and ≥7.00 mmol/L). A *P*-value for interaction was calculated.

Finally, restrictive Cubic Spline (RCS) function was used to graphically display the hazard ratios representing the dose-response association between IgG and the risk of pancreatic cancer. We used knots located at the 5, 50, and 95th percentiles. This analysis was performed using the RCS_RegSAS Macro created by Desquibet and Mariotti (45). All statistical analyses were conducted with Statistical Analysis Systems (SAS) release 9.4 (SAS Institute, Cary, NC).

## Results

### Immunoglobulin G

Characteristics of study participants with a measurement of IgG are shown in Table 1. During a mean follow-up of 21.3 years, 689 participants developed pancreatic cancer. The mean age at measurement in participants who later developed pancreatic cancer was higher (55.8 years) than in participants without pancreatic cancer (47.1 years).

**Table 1:** Descriptive statistics of study population with a measurement of IgG.

	**Pancreatic cancer *N* = 689 *n* (%)**	**No pancreatic cancer *N* = 135,532 *n* (%)**
**Mean Age (years) (SD)**	55.8 (11.37)	47.1 (14.33)
<55	319 (46.30)	95,342 (70.35)
≥55	370 (53.70)	40,190 (29.65)
**Gender**		
Men	388 (56.31)	68,995 (50.91)
Women	301 (43.69)	66,537 (49.09)
**SES**		
Unclassified/missing	74 (10.74)	13,561 (10.01)
Low	283 (41.07)	59,611 (43,98)
High	332 (48.19)	62,360 (46.01)
**Education**		
Missing	109 (15.82)	8,594 (6.34)
Low	211 (30.62)	37,876 (27.95)
Middle	241 (34.98)	55,617 (41.04)
High	128 (18.58)	33,445 (24.68)
**Charlson Comorbidity Index**		
0	622 (90.28)	126,594 (93.41)
1	49 (7.11)	6,265 (4.62)
2	13 (1.89)	1,565 (1.15)
3+	5 (0.73)	1,108 (0.82)
**Mean follow-up time (years) (SD)**	13.1 (7.64)	21.3 (6.67)
**Glucose (mmol/L)**		
Mean (SD)	5.34 (1.71)	5.01 (1.28)
<5.60 mmol/L	501 (72.71)	107,538 (79.35)
5.60–6.99 mmol/L	107 (15.53)	14,352 (10.59)
≥7.00 mmol/L	48 (6.97)	4,040 (2.98)
Missing	33 (4.79)	9,602 (7.08)
**IgG (g/L)**		
Mean (SD)	11.17 (2.77)	11.55 (3.03)
<6.10 g/L	14 (2.03)	1,493 (1.10)
6.10–14.99 g/L	601 (87.23)	116,704 (86.11)
≥15.00 g/L	74 (10.74)	17,335 (12.79)

Multivariate Cox regression (adjusted for age, gender, education, CCI, and serum glucose level) for the association between immunoglobulin G and the risk of pancreatic cancer showed that, compared to the IgG reference level of 6.10–14.99 g/L, there was a positive association for those with IgG levels <6.10 g/L [HR: 1.69 (95% CI 0.99–2.87)]. We also found an inverse association for those with IgG levels ≥15.00 g/L [0.82 (95% CI 0.64–1.05); *P*trend = 0.027] (Table 2). A sensitivity analysis to assess reverse causation by excluding those with follow-up time <1 year and <3 year did not affect the above findings (results not shown).

**Table 2:** Hazard ratio (HR) for risk of pancreatic cancer, in the study population with a measurement of IgG, with 95% confidence intervals (CI) using Cox proportional hazards model.

	**Pancreatic cancer/total *N***	**Hazard ratio^a^ (95% CI)**
**IgG (g/L)**		
<6.10 g/L	14/1,507	1.69 (0.99–2.87)
6.10–14.99 g/L	601/117,305	1.00 (ref)
≥15.00 g/L	74/17,409	0.82 (0.64–1.05)
*P*-value for trend		0.027

a *Adjusted for age, gender, education, CCI, and serum glucose (continuous variable)*.

**Figure 1:**
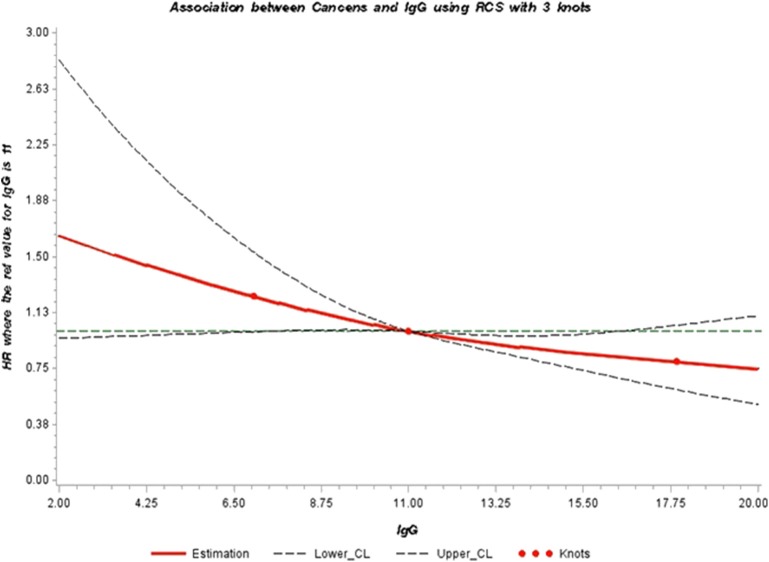
Adjusted dose-response association between serum levels of IgG (g/L) and risk of pancreatic cancer (HR) using restrictive cubic splines. The direction of the hazard ratios observed in Tables 2, 3 was consistent with the shape of the curve, which presents a positive association for IgG levels lower than 11.00 g/L with pancreatic cancer risk (HR > 1.00) and a protective effect for high levels of IgG (11.00–20.00 g/L) (HR <1.00). However, the inverse association was only statistically significant for concentrations of IgG between 11.00 and 16.00 g/L.

Stratified analysis by gender showed a similar inverse association between categories of IgG and the risk of developing pancreatic cancer. However, the strength of the association was more pronounced for women (P = 0.003) (Table 3). No effect modification by age and glucose levels was observed (results not shown). We further illustrated the association between serum IgG and the risk of developing pancreatic cancer through a dose-response curve with restrictive cubic splines (Figure 1). The direction of the hazard ratios observed in Tables 2, 3 was consistent with shape of the curve, which presented a positive association for IgG levels lower than 11.00 g/L with pancreatic cancer risk and a protective effect for high levels of IgG (11.00−20.00 g/L).

**Table 3:** Hazard ratio (HR) for risk of pancreatic cancer stratified by gender, in the study population with a measurement of IgG, with 95% confidence intervals from Cox proportional Hazards model.

	**Hazard Ratio^a^ (95% CI)**	
**Gender**	**Male**	**Female**
Pancreatic cancer/total (n)	388/69,383	301/66,838
IgG (g/L)		
<6.10 g/L	1.46 (0.65–3.27)	1.98 (0.97–4.00)
6.10–14.99 g/L	1.00 (ref)	1.00 (ref)
≥15.00 g/L	0.95 (0.69–1.29)	0.64 (0.43–0.97)
*P*-value for interaction	0.003	

a *Adjusted for age, education, CCI, and serum glucose (continuous variable)*.

### Immunoglobulin A and Immunoglobulin M

Characteristics of study participants with a measurement of IgA are shown in Table 4. During a mean follow-up of 16.5 years, 169 participants developed pancreatic cancer. Characteristics of study participants with a measurement of IgM are shown in Table 5. During a mean follow-up of 15.4 years, 117 participants developed pancreatic cancer.

**Table 4:** Descriptive statistics of study population with a measurement of IgA.

	**Pancreatic cancer *N* = 169 *n* (%)**	**No pancreatic cancer *N* = 41,731 *n* (%)**
**Mean Age (years) (SD)**	60.3 (12.07)	49.6 (16.10)
<55	58 (34.32)	27,142 (65.04)
≥55	111 (65.68)	14,589 (34.96)
**Gender**		
Men	69 (40.83)	14,999 (35.94)
Women	100 (59.17)	26,732 (64.06)
**SES**		
Unclassified/Missing	27 (15.98)	7,499 (17.97)
Low	69 (40.83)	18,137 (43.46)
High	73 (43.20)	16,095 (38.57)
**Education**		
Missing	14 (8.28)	2,358 (5.65)
Low	50 (29.59)	10,787 (25.85)
Middle	64 (37.87)	17,734 (42.50)
High	41 (24.26)	10,852 (26.00)
**Charlson Comorbidity Index**		
0	138 (81.66)	37,290 (89.36)
1	22 (13.02)	2,950 (7.07)
2	7 (4.14)	856 (2.05)
3+	2 (1.18)	635 (1.52)
**Mean follow-up time (years) (SD)**	8.8 (5.88)	16.5 (5.22)
**Glucose (mmol/L)**		
Mean (SD)	5.50 (1.76)	5.08 (1.42)
<5.60 mmol/L	96 (56.80)	25,481 (61.06)
5.60–6.99 mmol/L	25 (14.79)	3,990 (9.56)
≥7.00 mmol/L	11 (6.51)	1,315 (3.15)
Missing	37 (21.89)	10,945 (26.23)
**IgA (g/L)**		
Mean (SD)	2.47 (1.20)	2.32 (1.20)
<3.66 g/L	151 (89.35)	37,228 (89.21)
≥3.66 g/L	18 (10.65)	4,503 (10.79)

**Table 5:** Descriptive statistics of study population with a measurement of IgM.

	**Pancreatic cancer *N* = 117 *n* (%)**	**No pancreatic cancer *N* = 29,802 *n* (%)**
**Mean Age (years) (SD)**	60.9 (11.76)	50.8 (16.25)
<55	36 (30.77)	18,613 (62.46)
≥55	81 (69.23)	11,189 (37.54)
**Gender**		
Men	48 (41.03)	10,852 (36.41)
Women	69 (58.97)	18,950 (63.59)
**SES**		
Unclassified/missing	18 (15.38)	5,675 (19.04)
Low	51 (43.59)	12,759 (42.81)
High	48 (41.03)	11,368 (38.15)
**Education**		
Missing	8 (6.84)	1,658 (5.56)
Low	37 (31.62)	7,885 (26.46)
Middle	43 (36.75)	12,585 (42.23)
High	29 (24.79)	7,674 (25,75)
**Charlson comorbidity index**		
0	88 (75.21)	26,205 (87.93)
1	20 (17.09)	2,349 (7.88)
2	6 (5.13)	697 (2.34)
3+	3 (2.56)	551 (1.85)
**Mean follow-up time (years) (SD)**	8.15 (5.53)	15.4 (4.73)
**Glucose (mmol/L)**		
Mean (SD)	5.53 (1.59)	5.19 (1.51)
<5.60 mmol/L	60 (51.28)	16,259 (54.56)
5.60–6.99 mmol/L	19 (16.24)	3,140 (10.54)
≥7.00 mmol/L	8 (6.84)	1,046 (3.51)
Missing	30 (25.64)	9,357 (31.40)
**IgM (g/L)**		
Mean (SD)	1.11 (0.72)	1.28 (0.96)
<1.40 g/L	90 (76.92)	20,330 (68.22)
≥1.40 g/L	27 (23.08)	9,472 (31.78)

We found no association between immunoglobulin A or immunoglobulin M and the risk of pancreatic cancer (Tables 6, 7).

**Table 6:** Hazard ratio (HR) for risk of pancreatic cancer, in the study population with a measurement of IgA, with 95% confidence intervals (CI) using Cox proportional hazards model.

	**Pancreatic cancer/total *N***	**Hazard ratio^a^ (95% CI)**
**IgA (g/L)**		
<3.66 g/L	151/37,379	1.00 (ref)
≥3.66 g/L	18/4,521	0.70 (0.39–1.26)

a *Adjusted for age, education, CCI, and serum glucose (continuous variable)*.

**Table 7:** Hazard ratio (HR) for risk of pancreatic cancer, in the study population with a measurement of IgM, with 95% confidence intervals (CI) using Cox proportional hazards model.

	**Pancreatic cancer/total *N***	**Hazard ratio^a^ (95% CI)**
**IgM (g/L)**		
<1.40 g/L	90/20,420	1.00 (ref)
≥1.40 g/L	27/9499	0.82 (0.50–1.35)

a *Adjusted for age, education, CCI, and serum glucose (continuous variable)*.

## Discussion

In this project, we indicate the presence of an inverse association between serum IgG levels and the risk of developing pancreatic cancer in the Swedish AMORIS cohort. The inverse association was stronger in females compared with males. Pre-diagnostic serum levels of IgA or IgM did not show any association with the risk of pancreatic cancer.

The immune system has been thought to modulate the development and evolution of pancreatic cancer (46). Tobacco smoking, chronic pancreatitis, obesity and long-standing diabetes are established risk factors for pancreatic cancer (4, 5, 47, 48). All these risk factors can influence the immune response, induce the formation of pre-malignant lesions and stimulate pancreatic cancer development (49, 50). Pancreatic cancer tissue contains multiple immunosuppressive cell types, suggesting an impairment of the immune response in the tumor micro-environment (50). Evidence is growing for the role of the tumor-infiltrating B cells in the initiation and progression of pancreatic cancer (51). Tanaka et al., among other groups, also identified high serum levels of IgG on advanced pancreatic patients indicating IgG as a potential diagnostic test for pancreatic cancer (52).

Recent experimental and epidemiological data suggest the importance of the microbiome in pancreatic cancer development and its potential use as a marker of disease susceptibility (9, 49, 53, 54). Kau et al. highlighted the close interplay between life-style factors, such as diet and nutritional status, with the microbial ecology in the humans digestive system and its modulation of the immune system (55). Moreover, there is increasing evidence of the influence of the microbiota in the development of human diseases such as obesity and diabetes, established risks factors for pancreatic cancer (56, 57). More specifically, exposure to the bacterium *Helicobacter Pylori* is suggested as a risk factor for pancreatic cancer (14–17). Data also suggest a positive association between periodontal disease, due to an infection of the periodontal bacterium *Porphyromonas gingivalis and Fusobacterium species*, and pancreatic cancer risk (8, 18–23). The microbiota immune modulation promoting cancer pathogenesis has been explained through inflammatory processes in the tumor tissue, however it has also been shown in mice that bacteria not present in the tumorigenic tissue can promote carcinogenesis, nor do they need to cause inflammation in the tumor microenvironment (8, 58). New studies should further explore the interplay between lifestyle factors, microbiota and dysregulation of the immune system, which could provide new avenues to better understand the etiology of pancreatic cancer.

To our knowledge, this study is the first cohort that prospectively evaluate the association between pre-diagnostic serum markers of the overall humoral immune response and risk of pancreatic cancer. The observed inverse association between serum IgG and the risk of developing pancreatic cancer is consistent with the work by Michaud et al. (20): in a cluster analysis on oral bacteria antibodies, the cluster with overall higher levels of antibodies against 25 oral bacteria had a 45% lower risk of pancreatic cancer development than a cluster with overall lower levels of antibodies. This finding suggest that higher levels of antibodies against oral bacteria may reflect a stronger immune status which could have beneficial impact on reducing the risk of pancreatic cancer (20). Moreover, experimental studies found a similar protective effect for increased levels of IgG. Hamanaka et al., found circulating IgG antibodies to be a favorable prognostic factor for pancreatic cancer (59). In a recent publication, Follia et al. presented novel metabolic subtypes in pancreatic cancer and observed that the high immune infiltrated tumors had a better prognosis (60).

Regarding the stronger inverse association observed between IgG levels and pancreatic cancer, presented in women in our study, we suggest that this may indicate that sex is an effect modifier in this association given the differential distribution of immunoglobulin levels between sex that has been described previously (43).

The Swedish AMORIS cohort is one of the largest prospective cohort studies with detailed information on a range of serum biomarkers, including large number of baseline measurements of serum markers of the humoral immune system, all measured at the same clinical laboratory (61) at baseline health examinations during the period 1985–1996. The cohort has a 30+ year follow-up in Swedish national registers, including the Cancer, Patient, Census and Cause-of-Death registers, as well as quality registers for specific cancer sites. All participants of the AMORIS study were included by analysing blood and/or urine samples from health check-ups in non-hospitalized persons (62). However, any healthy cohort effect would not affect the internal validity of our study. The external validity of the study is not compromised given that the cancer incidence in the AMORIS population is comparable to the reported incidence in the Swedish population (35).

It was a limitation that immunoglobulin E (IgE), a serum biomarker of allergies studied previously in relation with pancreatic cancer (63), was only measured for a small subset of participants, so that it could not be assessed in relation to risk of pancreatic cancer. In addition, repeated measurements would have been helpful to verify the lag time between changes in markers of the humoral immune system and risk of pancreatic cancer. However, most of the individuals did not have multiple measurements. Information on other possible confounders such as BMI and smoking status was not available on AMORIS, therefore all models were adjusted for Charlson Comorbidity Index as a proxy for lifestyle factors. Biological material was unfortunately not available for further analyses. Molecular pathological analyses, such as the presence of immune cell infiltrates on the tumor tissue samples, can be an important resource in future studies to explore the potential association of the immune system, microbiome, lifestyle factors and pancreatic cancer development and to validate the epidemiological findings.

## Conclusion

To our knowledge, this study is the first prospective cohort study evaluating the association between pre-diagnostic serum markers of the overall humoral immune response and the risk of pancreatic cancer. We observed an inverse association between pre-diagnostic serum levels of IgG and the risk of pancreatic cancer. Our findings highlight the need to investigate the roles of different components of humoral immunity and agents that may cause a humoral immune response related to pancreatic cancer. Future studies could provide further insight into potential biological mechanisms by exploring longitudinal data such as repeated measurements of pre-diagnostic serum markers of the humoral immune system. Moreover, molecular pathological epidemiological studies, exploring tumor tissues, can potentially untangle the interlink between lifestyle factors, microbiome, carcinogenesis and the immune system (64–66).

## Data Availability Statement

Access to data for collaboration is provided by the Steering group members of the AMORIS study by request in email under the heading AMORIS Cohort Collaboration. This can be found at the AMORIS homepage http://amoriscohort.imm.ki.se.

## Ethics Statement

This study complied with the Declaration of Helsinki and was approved by the Ethics Review Board of the Karolinska Institute who waived the need for consent.

## Author Contributions

SS, AS, NH, GW, and HG: data collection. SS, AS, DM, NH, GW, HG, LH, IJ, and MV: data analysis and interpretation. SS and AS: draft of manuscript. SS, DM, AS, DS, SK, DJ, NH, GW, HG, LH, IJ, and MV: final editing of manuscript. We can confirm that the manuscript has been read and approved by all named authors and that there are no other persons who satisfied the criteria for authorship but are not listed.

### Conflict of Interest

The authors declare that the research was conducted in the absence of any commercial or financial relationships that could be construed as a potential conflict of interest.

## References

[1] Rahib L Smith BD Aizenberg R Rosenzweig AB Fleshman JM Matrisian LM. Projecting cancer incidence and deaths to 2030: the unexpected Burden of thyroid, liver, and pancreas cancers in the Untied States. Cancer Res. (2014). 74:2913–21. 10.1158/0008-5472.CAN-14-0155 24840647

[2] Luo G Zhang Y Guo P Ji H Xiao Y Li K. Global patterns and trends in pancreatic cancer incidence: age, period, and birth cohort analysis. Pancreas. (2019) 48:199–208. 10.1097/MPA.0000000000001230 30589831

[3] Lynch SM Vrieling A Lubin JH Kraft P Mendelsohn JB Hartge P. Cigarette smoking and pancreatic cancer: a pooled analysis from the pancreatic cancer cohort consortium. Am J Epidemiol. (2009) 170:403–13. 10.1093/aje/kwp134 19561064 PMC2733861

[4] Ben Q Xu M Ning X Liu J Hong S Huang W. Diabetes mellitus and risk of pancreatic cancer: a meta-analysis of cohort studies. Eur J Cancer. (2011) 47:1928–37. 10.1016/j.ejca.2011.03.003 21458985

[5] Duell EJ Lucenteforte E Olson SH Bracci PM Li D Risch HA. Pancreatitis and pancreatic cancer risk: a pooled analysis in the International Pancreatic Cancer Case-Control Consortium (PanC4). Ann Oncol. (2012) 23:2964–70. 10.1093/annonc/mds140 22767586 PMC3477881

[6] Ekbom A Trichopoulos D Pancreatic cancer. In: Adami HO, Hunter D, Trichopoulos D, editors. Textbook of Cander Epidemiology. New York, NY: Oxford University Press (2008). p. 333–48. 10.1093/acprof:oso/9780195311174.003.0013

[7] Ghaneh P Costello E Neoptolemos JP. Biology and management of pancreatic cancer. Gut. (2007) 56:1134–52. 10.1136/gut.2006.113068 17625148 PMC1955499

[8] Michaud DS. Role of bacterial infections in pancreatic cancer. Carcinogenesis. (2013) 34:2193–7. 10.1093/carcin/bgt249 23843038 PMC3786384

[9] Wang C Li J. Pathogenic microorganisms and pancreatic cancer. Gastrointestinal Tumors. (2015) 2:41–7. 10.1159/000380896 26673459 PMC4668790

[10] Tomasiewicz K Modrzewska R Lyczak A Krawczuk G TT virus infection and pancreatic cancer: relationship or accidental coexistence. World J Gastroenterol. (2005) 11:2847 10.3748/wjg.v11.i18.2847 15884138 PMC4305932

[11] Xu JH Fu JJ Wang XL Zhu JY Ye XH Chen SD Hepatitis B or C viral infection and risk of pancreatic cancer: a meta-analysis of observational studies. World J Gastroenterol. (2013) 19:4234–41. 10.3748/wjg.v19.i26.4234 23864789 PMC3710428

[12] Wang Y Yang S Song F Cao S Yin X Xie J. Hepatitis B virus status and the risk of pancreatic cancer: a meta-analysis. Eur J Cancer Prev. (2013) 22:328–34. 10.1097/CEJ.0b013e32835b6a21 23165286

[13] Desai R Patel U Sharma S Singh S Doshi S Shaheen S. Association between hepatitis B infection and pancreatic cancer: a population-based analysis in the United States. Pancreas. (2018) 47:849–55. 10.1097/MPA.0000000000001095 29939908

[14] Schulte A Pandeya N Fawcett J Fritschi L Risch HA Webb PM. Association between Helicobacter pylori and pancreatic cancer risk: a meta-analysis. Cancer Causes Cont. (2015) 26:1027–35. 10.1007/s10552-015-0595-3 25951801

[15] Raderer M Wrba F Kornek G Maca T Koller DY Weinlaender G. Association between Helicobacter pylori infection and pancreatic cancer. Oncology. (1998) 55:16–9. 10.1159/000011830 9428370

[16] Stolzenberg-Solomon RZ Blaser MJ Limburg PJ Perez-Perez G Taylor PR Virtamo J. Helicobacter pylori seropositivity as a risk factor for pancreatic cancer. J Nat Cancer Inst. (2001) 93:937–41. 10.1093/jnci/93.12.937 11416115

[17] Xiao M Wang Y Gao Y. Association between Helicobacter pylori infection and pancreatic cancer development: a meta-analysis. PLoS ONE. (2013) 8:e75559. 10.1371/journal.pone.0075559 24086571 PMC3784458

[18] Liu X-b Gao Z-y Sun C-t Wen H Gao B Li S-b. The potential role of *P. gingivalis* in gastrointestinal cancer: a mini review. Infect Agents Cancer. (2019) 14:23. 10.1186/s13027-019-0239-4 31516546 PMC6734237

[19] Ahn J Segers S Hayes RB. Periodontal disease, *Porphyromonqs gingivalis* serum antibody levels and orodigestive cancer mortality. Carcinogenesis. (2012) 33:1055–8. 10.1093/carcin/bgs112 22367402 PMC3334514

[20] Michaud DS Izard J Wilhelm-Benartzi CS You DH Grote VA Tjønnel A. Plasma antibodies to oral bacteria and risk of pancreatic cancer in a large European prospective cohort study. Gut. (2013) 62:1764–70. 10.1158/1538-7445.AM2012-LB-328 22990306 PMC3815505

[21] Hujoel PP Drangsholt M Spiekerman C Weiss NS. An exploration of the Periodontotis-cancer association. Ann Epidemiol. (2003) 13:312–6. 10.1016/S1047-2797(02)00425-8 12821269

[22] Michaud DS Joshipura K Giovannucci E Fuchs CS. A prospective study of periodontal disease and pancreatic cancer in US male health professionals. J Natl Cancer Inst. (2007) 99:171–5. 10.1093/jnci/djk021 17228001

[23] Mitsuhashi K Nosho K Sukawa Y Matsunaga Y Ito M Kurihara H. Association of Fusobacterium species in pancreatic cancer tissues with molecular features and prognosis. Oncotarget. (2015) 6:7209–20. 10.18632/oncotarget.3109 25797243 PMC4466679

[24] Michaud DS Fu Z Shi J Chung M. Periodontal disease, tooth loss, and cancer risk. Epidemiol Rev. (2017) 39:49–58. 10.1093/epirev/mxx006 28449041 PMC5868279

[25] Cotterchio M Lowcock E Hudson TJ Greenwood C Gallinger S. Association between allergies and risk of pancreatic cancer. Cancer Epidemiol Biomark Prev. (2014) 23:469–80. 10.1158/1055-9965.EPI-13-0965 24554712 PMC3951672

[26] Merrill RM Isakson RT Beck RE. The association between allergies and cancer: what is currently known? Ann Allergy Asthma Immunol. (2007) 99:102–17. 10.1016/S1081-1206(10)60632-1 17718097

[27] Turner MC Chen Y Krewski D Ghadirian P. An overview of the association between allergy and cancer. Int J Cancer. (2006) 118:3124–32. 10.1002/ijc.21752 16395696

[28] Wulaningsih W Holmberg L Garmo H Karagiannis SN Ahlstedt S Malmstrom H. Investigating the association between allergen-specific immunoglobulin E, cancer risk and survival. Oncoimmunology. (2016) 5:e1154250. 10.1080/2162402X.2016.1154250 27471625 PMC4938379

[29] Gandini S Lowenfels AB Jaffee EM Armstrong TD Maisonneuve P. Allergies and the risk of pancreatic cancer: a meta-analysis with review of epidemiology and biological mechanisms. Cancer Epidemiol Biomark Prev. (2005) 14:1908–16. 10.1158/1055-9965.EPI-05-0119 16103436

[30] Ingvarsson J Wingren C Carlsson A Ellmark P Wahren B Engström G. Detection of pancreatic cancer using antibody microarray-based serum protein profiling. Proteomics. (2008) 8:2211–9. 10.1002/pmic.200701167 18528842

[31] Orchekowski R Hamelinck D Li L Gliwa E VanBrocklin M Marrero JA. Antibody microarray profiling reveals individual and combined serum proteins associated with pancreatic cancer. Cancer Res. (2005) 65:11193–202. 10.1158/0008-5472.CAN-05-1436 16322270

[32] Kamisawa T Chen PY Tu Y Nakajima H Egawa N Tsuruta K. Pancreatic cancer with a high serum IgG4 concentration. World J Gastroenterol. (2006) 12:6225. 10.3748/wjg.v12.i38.6225 17036401 PMC4088123

[33] Kotera Y Fontenot JD Pecher G Metzgar RS Finn OJ. Humoral immunity against a tandem repeat epitope of human mucin MUC-1 in sera from breast, pancreatic, and colon cancer patients. Cancer Res. (1994) 54:2856–60. 7514493

[34] Talar-Wojnarowska R Gasiorowska A Olakowski M Dranka-Bojarowska D Lampe P Smigielski J. Utility of serum IgG, IgG4 and carbonic anhydrase II antibodies in distinguishing autoimmune pancreatitis from pancreatic cancer and chronic pancreatitis. Adv Med Sci. (2014) 59:288–92. 10.1016/j.advms.2014.08.003 25194335

[35] Walldius G Malmström H Jungner I de Faire U Lambe M Van Hemelrijck M. Cohort profile: the AMORIS cohort. Int J Epidemiol. (2017) 46:1103–1103i. 10.1093/ije/dyw333 28158674

[36] Walldius G Jungner I Holme I Aastveit AH Kolar W Steiner E. High apolipoprotein B, low apolipoprotein A-I and improvement in the prediction of fatal myocardial infarction (AMORIS study):a prospective study. Lancet. (2001) 358:2026–33. 10.1016/S0140-6736(01)07098-2 11755609

[37] Holme I Aastveit AH Jungner I Walldius G. Relationships between lipoprotein components and risk of myocardial infarction: age, gender and short versus longer follow-up periods in the Apolipoprotein MOrtality RISk study (AMORIS). J Int Med. (2008) 264:30–8. 10.1111/j.1365-2796.2008.01925.x 18298486

[38] Holme I Aastveit AH Hammar N Jungner I Walldius G. Relationships between lipoprotein components and risk of ischaemic and haemorrhagic stroke in the Apolipoprotein MOrtality RISk study (AMORIS). J Int Med. (2009) 265:275–87. 10.1111/j.1365-2796.2008.02016.x 19019184

[39] Walldius G Jungner I Kolar W Holme I Steiner E. High cholesterol and triglyceride values in Swedish males and females: increased risk of fatal myocardial infarction. *First* report from the AMORIS (Apolipoprotein related MOrtality RISk) study. Blood Pres Suppl. (1992) 4:35–42. 1345333

[40] Junger I Kolar W CALAB. S-Immunoglobulin A (1994).

[41] Junger I Kolar W CALAB. S-Immunoglobulin G (1994).

[42] Junger I Kolar W CALAB. S-Immunoglobulin M (1994).

[43] Gonzalez-Quintela A Alende R Gude F Campos J Rey J Meijide LM. Serum levels of immunoglobulins (IgG, IgA, IgM) in a general adult population and their relationship with alcohol consumption, smoking and common metabolic abnormalities. Clin Exp Immunol. (2007) 151:42–50. 10.1111/j.1365-2249.2007.03545.x 18005364 PMC2276914

[44] Wians FH Normal laboratory values: blood, plasma, and serum. MSD Manual. (2018).

[45] Desquilbet L Mariotti F. Dose-response analyses using restricted cubic spline functions in public health research. Stat Med. (2010) 29:1037–57. 10.1002/sim.3841 20087875

[46] Grote VA Kaaks R Nieters A Tjønnel A Halkjær J Overvad K. Inflammation marker and risk of pancreatic cancer: a nested case-control study within the EPIC cohort. Br J Cancer. (2012) 106:1866–74. 10.1038/bjc.2012.172 22617158 PMC3364108

[47] Andersen DK Andren-Sandberg A Duell EJ Goggins M Korc M Petersen GM. Pancreatitis-diabetes-pancreatic cancer: summary of an NIDDK-NCI workshop. Pancreas. (2013) 42:1227–37. 10.1097/MPA.0b013e3182a9ad9d 24152948 PMC3878448

[48] Raimondi S Lowenfels AB Morselli-Labate AM Maisonneuve P Pezzilli R. Pancreatic cancer in chronic pancreatitis; aetiology, incidence, and early detection. Best Pract Res Clin Gastroenterol. (2010) 24:349–58. 10.1016/j.bpg.2010.02.007 20510834

[49] Michaud DS Izard J. Microbiota, oral microbiome, and pancreatic cancer. Cancer J. (2014) 20:203–6. 10.1097/PPO.0000000000000046 24855008 PMC4160879

[50] Inman KS Francis AA Murray NR. Complex role for the immune system in initiation and progression of pancreatic cancer. Wotld J Gastroenterol. (2014) 20:11160–81. 10.3748/wjg.v20.i32.11160 25170202 PMC4145756

[51] Roghanian A Fraser C Kleyman M Chen J. B cells promote pancreatic tumorigenesis. Cancer Discov. (2016) 6:230–2. 10.1158/2159-8290.CD-16-0100 26951836

[52] Tanaka M Komatsu N Yanagimoto Y Oka M Shichijo S Okuda S. Development of a new diagnostic tool for pancreatic cancer: simultaneous measurement of antibodies against peptides recognized by cytotoxic T lymphocytes. Kurume Med J. (2006). 53:63–70. 10.2739/kurumemedj.53.63 17317934

[53] Archibugi L Signoretti M Capurso G. The microbiome and pancreatic cancer: an evidence-based association? Clin Gastroenterol. (2018) 52:S82–5. 10.1097/MCG.0000000000001092 30001289

[54] Wei M-Y Shi S Liang C Meng Q-C Hua J Zhang Y-Y. The microbiota and microbiome in pancreatic cancer: more influential than expected. Mol Cancer. (2019) 18:97. 10.1186/s12943-019-1008-0 31109338 PMC6526613

[55] Kau AL Ahern PP Griffin NW Goodman AL Gordon JI. Human nutrition, the gut microbiome and the immune system. Nature. (2011) 474:327. 10.1038/nature10213 21677749 PMC3298082

[56] Shen J Obin MS Zhao L. The gut microbiota, obesity and insulin resistance. Mol Aspects Med. (2013) 34:39–58. 10.1016/j.mam.2012.11.001 23159341

[57] Qin J Li Y Cai Z Li S Zhu J Zhang F. A metagenome-wide association study of gut microbiota in type 2 diabetes. Nature. (2012) 490:55. 10.1038/nature11450 23023125

[58] Fox JG Feng Y Theve EJ Raczynski AR Fiala JL Doernte AL. Gut microbes define liver cancer risk in mice exposed to chemical and viral transgenic hepatocarcinogens. Gut. (2010) 59:88–97. 10.1136/gut.2009.183749 19850960 PMC3891362

[59] Hamanaka Y Suehiro Y Fukui M Shikichi K Imai K Hinoda Y. Circulating anti-MUC1 IgG antibodies as a favorable prognostic factor for pancreatic cancer. Int J Cancer. (2003) 103:97–100. 10.1002/ijc.10801 12455059

[60] Follia L Ferrero G Mandili G Beccuti M Giordano D Spadi R. Integrative analysis of novel metabolic subtypes in pancreatic cancer fosters new prognostic biomarkers. Front Oncol. (2019) 9:115. 10.3389/fonc.2019.00115 30873387 PMC6400843

[61] Holme I Aastveit AH Hammar N Jungner I Walldius G. Inflammatory markers, lipoprotein components and risk of major cardiovascular events in 65,005 men and women in the Apolipoprotein MOrtality RISk study (AMORIS). Atherosclerosis. (2013) 213:299–305. 10.1016/j.atherosclerosis.2010.08.049 20843515

[62] Wulaningsih W Michaelsson K Garmo H Hammar N Jungner I Walldius G. Serum calcium and risk of gastrointestinal cancer in the Swedish AMORIS study. BMC Public Health. (2013) 13:663. 10.1186/1471-2407-13-257 23866097 PMC3729677

[63] Fu SL Pierre J Smith-Norowitz TA Hagler M Bowne W Pincus MR. Immunoglobulin E antibodies from pancreatic cancer patients mediate antibody-dependent cell-mediated cytotoxicity against pancreatic cancer cells. Clin Exp Immunol. (2008) 153:401–9. 10.1111/j.1365-2249.2008.03726.x 18803764 PMC2527370

[64] Ogino S Nowak JA Hamada T DAM Jr Nishihara R. Insights into pathogenic interactions among environment, host, and tumor at the crossroads of molecular pathology and epidemiology. Annu Rev Pathol Mech Dis. (2019) 14:83–103. 10.1146/annurev-pathmechdis-012418-012818 30125150 PMC6345592

[65] Hamada T Nowak JA Milner DA Jr Song M Ogino S. Integration of microbiology, molecular pathology, and epidemiology: a new paradigm to explore the pathogenesis of microbiome-driven neoplasms. J Pathol. (2019) 247:615–28. 10.1002/path.5236 30632609 PMC6509405

[66] Ogino S Nowak JA Hamada T Phipps AI Peters U Milner DA Jr. Integrative analysis of exogenous, endogenous, tumour and immune factors for precision medicine. Gut. (2018) 67:1168–80. 10.1136/gutjnl-2017-315537 29437869 PMC5943183

